# Supplementary figures and images for: Neoplastic signatures: Comparative proteomics of canine hepatobiliary neuroendocrine tumors to normal niche tissue
Source: PLoS One. 2023 Jan 25;18(1):e0280928. doi: 10.1371/journal.pone.0280928 (PMC9876354; doi:10.1371/journal.pone.0280928)

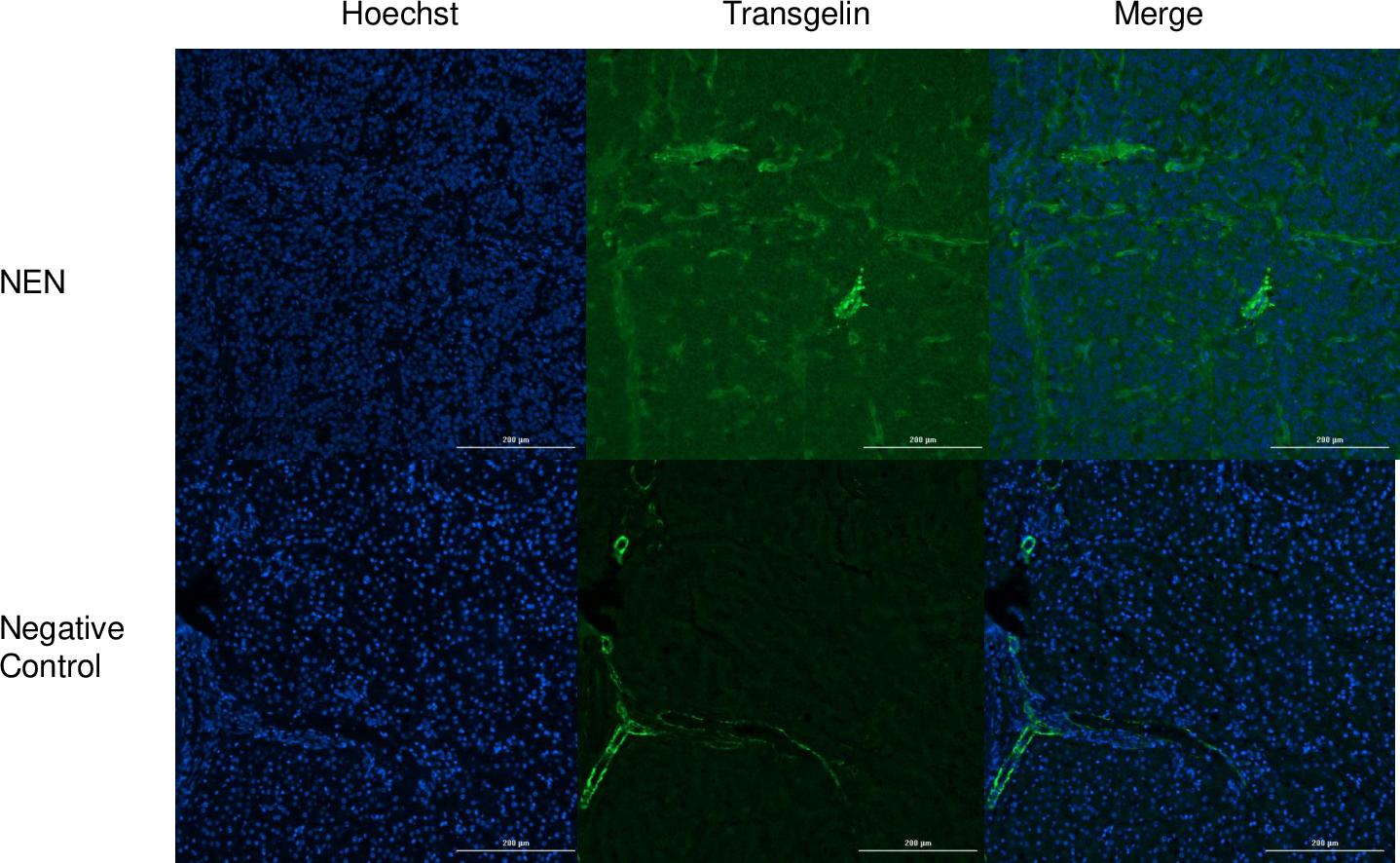

Supplement: S1 Fig — Nuclei are labeled by Hoescht stain (blue, left panel). Gall bladder neuroendocrine neoplasm demonstrates cytoplasmic positive transgelin (green, middle panel and right panel) immunofluorescence compared to negative control (normal canine liver), wherein only endothelium expresses transgelin. Scale bars: 200 micrometers. (TIF) [file pone.0280928.s001.tif]

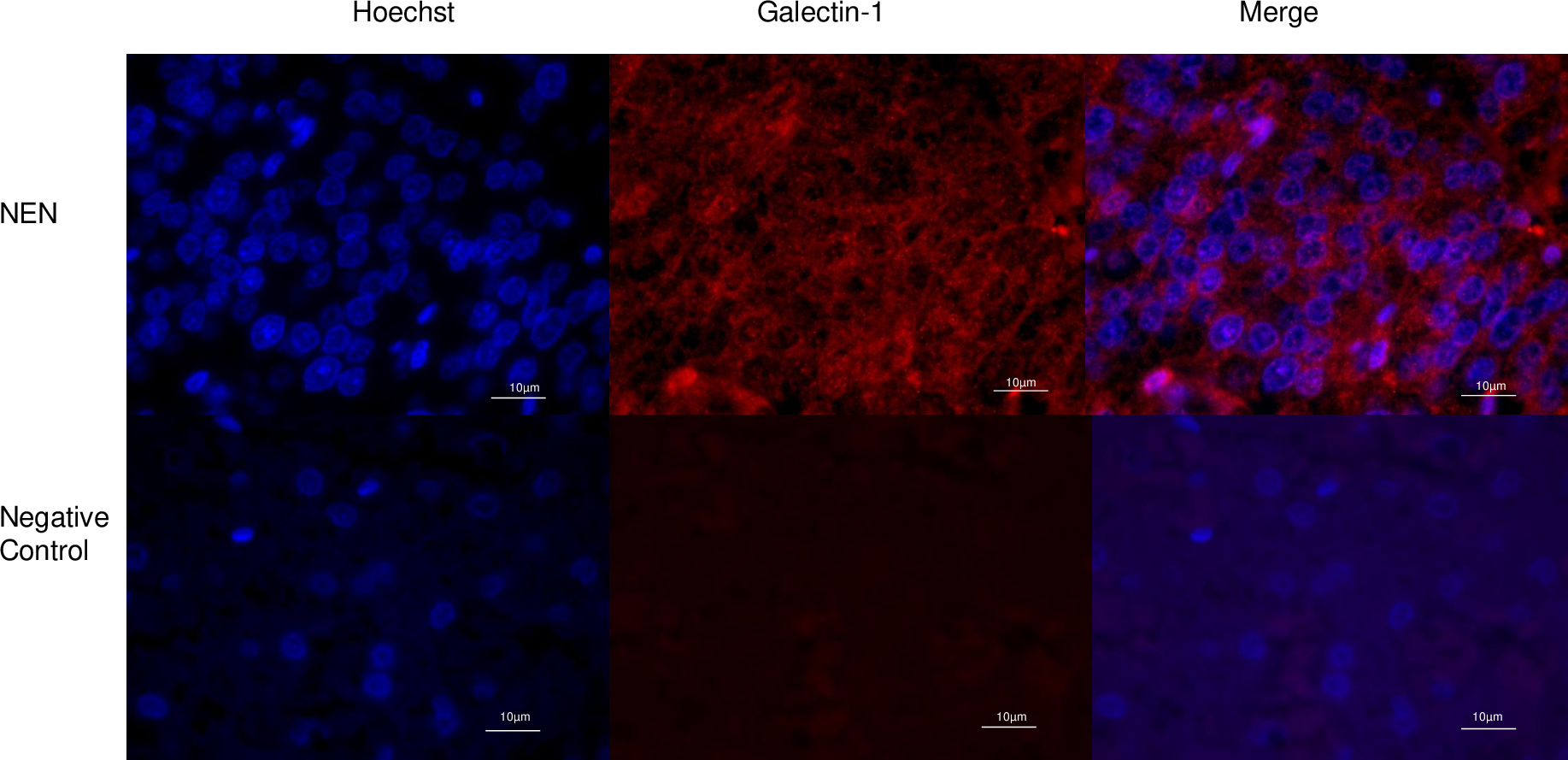

Supplement: S2 Fig — Nuclei are labeled by Hoescht stain (blue, left panels). Gall bladder neuroendocrine neoplasm demonstrates positive galectin-1 (red, middle and right top panels) immunofluorescence compared to negative control (normal canine kidney). Scale bars: 10 micrometers. (TIF) [file pone.0280928.s002.tif]
