# Supplementary material for: Neoplastic signatures: Comparative proteomics of canine hepatobiliary neuroendocrine tumors to normal niche tissue
Source: PLoS One. 2023 Jan 25;18(1):e0280928. doi: 10.1371/journal.pone.0280928 (PMC9876354; doi:10.1371/journal.pone.0280928)
Supplement: Attachment — Submitted filename: PONE-D-22-02231Reviewer comments.docx [file pone.0280928.s003.docx]

The author compared the proteomes of 3 canine hepatobiliary neoplasms to normal hepatobiliary tissue, which is an attractive topic, as hepatobiliary neuroendocrine neoplasm is rare cancers in humans and primary hepatobiliary neoplasm omics studies is still lacking. The authors extracted proteins from canine hepatobiliary neoplasms and performed mass spectrometry analysis. The data showed that 32 proteins were up-regulated and 126 proteins were down-regulated. Among them, 6 up-regulated proteins were associated with non-hepatobiliary neuroendocrine tumors, 16 proteins were associated with neuroendocrine signal transduction, and 26 down-regulated proteins were enriched in altered metabolic pathways of cancer. However, I do not think at the current stage the article has a potential for consideration in the journal for the publication.

1. For screening up-regulated and down-regulated proteins in canine hepatobiliary neoplasms, in order to increase the persuasiveness of the article, it is recommended to add some experiments to verify the expression of significantly increased proteins.

2. I suggest, the clinical information should be added, such as patient classification.

3. Please add scale in Figure 2.
