# Supplementary material for: Neoplastic signatures: Comparative proteomics of canine hepatobiliary neuroendocrine tumors to normal niche tissue
Source: PLoS One. 2023 Jan 25;18(1):e0280928. doi: 10.1371/journal.pone.0280928 (PMC9876354; doi:10.1371/journal.pone.0280928)
Supplement: Attachment — Submitted filename: Response to Reviewers.docx [file pone.0280928.s004.docx]

PONE-D-22-02231-R1

The authors appreciate the recommendations for improvement provided by the reviewers. Our responses are listed below each comment.

Journal Requirements:

When submitting your revision, we need you to address these additional requirements.

1. Please ensure that your manuscript meets PLOS ONE's style requirements, including those for file naming. The PLOS ONE style templates can be found at

<https://journals.plos.org/plosone/s/file?id=wjVg/PLOSOne_formatting_sample_main_body.pdf> and

[https://journals.plos.org/plosone/s/file?id=ba62/PLOSOne_formatting_sample_title_authors_affiliations.pdf](https://nam04.safelinks.protection.outlook.com/?url=https%3A%2F%2Fjournals.plos.org%2Fplosone%2Fs%2Ffile%3Fid%3Dba62%2FPLOSOne_formatting_sample_title_authors_affiliations.pdf&data=05%7C01%7Cjohnston1%40lsu.edu%7C826328eb57f34107e8ba08dad1bcc262%7C2d4dad3f50ae47d983a09ae2b1f466f8%7C0%7C0%7C638052906046922901%7CUnknown%7CTWFpbGZsb3d8eyJWIjoiMC4wLjAwMDAiLCJQIjoiV2luMzIiLCJBTiI6Ik1haWwiLCJXVCI6Mn0%3D%7C3000%7C%7C%7C&sdata=XzNxq%2BjI%2FROzb9k7zmeDj%2F%2FOFqj%2FldBQDMwwAXyNDgs%3D&reserved=0)

Response: The templates have been reviewed. The manuscript and files have been modified to meet PLOS ONE’s style requirements.

2. Thank you for stating the following in the Acknowledgments Section of your manuscript:

” This work was supported in part by NIH grant 1P20GM135000-01A1 and the Louisiana State University, Department of Veterinary Clinical Sciences CORP grant. Proteomic analysis was performed by the IDeA National Resource for Quantitative Proteomics, funded by the National Institute of General Medical Sciences (NIGMS) grant R24GM137786. This study was also supported by the Arkansas Children's Research Institute, the Arkansas Biosciences Institute, and the Center for Translational Pediatric Research funded under the National Institutes of Health National Institute of General Medical Sciences (NIH/NIGMS) grant P20GM121293 and the National Science Foundation Award No. OIA-1946391.”

We note that you have provided additional information within the Acknowledgements Section that is not currently declared in your Funding Statement. Please note that funding information should not appear in the Acknowledgments section or other areas of your manuscript. We will only publish funding information present in the Funding Statement section of the online submission form.

Please remove any funding-related text from the manuscript and let us know how you would like to update your Funding Statement. Currently, your Funding Statement reads as follows:

‘AJ - NIH grant 1P20GM135000-01A1 and the Louisiana State University, Department of Veterinary Clinical Sciences CORP grant

SB - Proteomic analysis was funded by the National Institute of General Medical Sciences (NIGMS) grant R24GM137786.

SB- Arkansas Children's Research Institute, the Arkansas Biosciences Institute, and the Center for Translational Pediatric Research funded under the National Institutes of Health National Institute of General Medical Sciences (NIH/NIGMS) grant P20GM121293 and the National Science Foundation Award No. OIA-1946391.”

Please include your amended statements within your cover letter; we will change the online submission form on your behalf.

Response: The funding related statements have been removed from the Acknowledgments section and the cover letter includes the funding information previously omitted from the funding statement. Thank you for changing the online submission on our behalf.

3. Please review your reference list to ensure that it is complete and correct. If you have cited papers that have been retracted, please include the rationale for doing so in the manuscript text, or remove these references and replace them with relevant current references. Any changes to the reference list should be mentioned in the rebuttal letter that accompanies your revised manuscript. If you need to cite a retracted article, indicate the article’s retracted status in the References list and also include a citation and full reference for the retraction notice.

Response: The reference list has been reviewed. It is complete and correct. No cited papers have been retracted.

**Comments to the Reviewers**

5.Review Comments to the Author

Please use the space provided to explain your answers to the questions above. You may also include additional comments for the author, including concerns about dual publication, research ethics, or publication ethics. (Please upload your review as an attachment if it exceeds 20,000 characters)

Reviewer #1: The author compared the proteomes of 3 canine hepatobiliary neoplasms to normal hepatobiliary tissue, which is an attractive topic, as hepatobiliary neuroendocrine neoplasm is rare cancers in humans and primary hepatobiliary neoplasm omics studies is still lacking. The authors extracted proteins from canine hepatobiliary neoplasms and performed mass spectrometry analysis. The data showed that 32 proteins were up-regulated and 126 proteins were down-regulated. Among them, 6 up-regulated proteins were associated with non-hepatobiliary neuroendocrine tumors, 16 proteins were associated with neuroendocrine signal transduction, and 26 down-regulated proteins were enriched in altered metabolic pathways of cancer. However, I do not think at the current stage the article has a potential for consideration in the journal for the publication.
1. For screening up-regulated and down-regulated proteins in canine hepatobiliary neoplasms, in order to increase the persuasiveness of the article, it is recommended to add some experiments to verify the expression of significantly increased proteins.

Response: Thank you for this recommendation to improve the persuasiveness of the manuscript. Upregulation of two proteins of interest, transgelin and galectin-1, were validated by immunofluorescence microscopy (S1 and S2 Figs). Transgelin and galectin-1 have been previously associated with human neuroendocrine tumors and thus provide proof of principle.

2. I suggest, the clinical information should be added, such as patient classification.

Response: In addition to patient signalment, which was originally included, the following clinical information has been added to the text (lines 114-120): “The Boston terrier was asymptomatic with a normal complete blood count and serum biochemistry. The Doberman pinscher presented for hematemesis after dietary indiscretion and had a mild elevation in GGT and cholesterol with moderate to marked elevations in ALP and ALT, respectively. No extrahepatobiliary masses were identified on abdominal ultrasound or during surgery. The patient with the liver NEN was a 12-year-old, male neutered Rat terrier with a history of lethargy, polyuria, polydipsia, and diarrhea. He had a normal complete blood count and moderate elevations of ALP and ALT on serum biochemistry.”

3. Please add scale in Figure 2.

Response: A scale bar has been added to Figure 2.

Reviewer #2: There is limited research on proteomics available in veterinary oncology. Furthermore, there are currently no studies evaluating proteomics in canine hepatobiliary neuroendocrine tumors. Thus, this manuscript begins to help fill the gaps in our understanding of these tumors. The introduction, materials and methods, results, and figures are complete and thorough.

I have one proposed revision for the discussion section. The limitations of utilizing FFPE samples as opposed to fresh frozen tissue samples should be addressed, as fresh frozen tissue is considered gold standard for comparative proteomics. Since FFPE samples are more common and overall reliable, I feel this information is still an important contribution to the current body of literature.

Reviewer: Thank you for this suggestion. A discussion of the limitations of using FFPE tissue compared to fresh tissue has been included (lines 359-367): “An additional limitation of this work is the use of FFPE samples for proteome analysis. While fresh frozen samples are the gold standard for comparative proteomics, these are not routinely collected from veterinary patients. The primary flaws associated with formalin fixation include formaldehyde induced protein cross-linking and amino acid modifications, which leads to difficulties with protein profiling. However, recent improvements in mass spectrometry workflows have enabled FFPE proteome maps comparable to those derived from fresh frozen tissue. This study demonstrates that archived FFPE canine tumor samples can be a rich resource for biomarker discovery.”
